# Multiple Discrimination and Its Consequences for the Mental Health of Ethnic Minorities

**DOI:** 10.1192/j.eurpsy.2022.65

**Published:** 2022-09-01

**Authors:** M. Schouler-Ocak, J. Moran

**Affiliations:** Charité – Universitätsmedizin Berlin, Psychiatric University Clinic At St. Hedwig Hospital, Berlin, Germany

## Abstract

Discrimination is a violation of human rights. The Universal Declaration of Human Rights proclaims in Art. 1 the equality of all human beings without distinction as to race, colour, sex, sexual orientation, religion, age or health. International law assigns three main characteristics to discrimination: disadvantageous treatment, based on unlawful grounds, and lack of reasonable and objective justification. Thus, it must be based on an unlawful characteristic: Ethnicity, religion, national or social origin, language, physical appearance, descent, gender, sexual orientation, age or disability. A growing body of literature has recognized health disparities and has investigated the relationship between discrimination and poor health outcomes. Ethnic minority groups across the world face social and psychological challenges linked to their minority status, often involving discrimination. Furthermore, cumulative exposure to racial discrimination has incremental negative long-term effects on the mental health of ethnic minority people.Studies that examine exposure to discrimination only at one point in time may underestimate the contribution of racism to poor health. Lower patient-centered care was associated with higher perceptions of discrimination, despite experiences of continuous discrimination or discrimination experienced as different types. Further, dissatisfaction with care was associated with discrimination, particularly when experienced in various forms. These findings reinforce a need for patient-provider communication that is inclusive and eliminates perceptions of discrimination and bias, increases patient-centeredness, and improves overall clinical care. Additionally, these results stress the need for more research investigating the relationship between discrimination and outcomes in patients, as perceived discrimination manifests as a significant barrier to effective disease management.

**Disclosure:**

No significant relationships.

